# Triple Monitoring May Avoid Intraneural Injection during Interscalene Brachial Plexus Block for Arthroscopic Shoulder Surgery: A Prospective Preliminary Study

**DOI:** 10.3390/jcm10040781

**Published:** 2021-02-16

**Authors:** Giuseppe Pascarella, Alessandro Strumia, Fabio Costa, Stefano Rizzo, Romualdo Del Buono, Luigi Maria Remore, Federica Bruno, Felice Eugenio Agrò

**Affiliations:** 1 Unit of Anaesthesia, Intensive Care and Pain Management, Department of Medicine, Università Campus Bio-Medico di Roma, Via Álvaro del Portillo 21, 00128 Rome, Italy; g.pascarella@unicampus.it (G.P.); f.costa@unicampus.it (F.C.); s.rizzo@unicampus.it (S.R.); l.remore@unicampus.it (L.M.R.); federica.bruno@unicampus.it (F.B.); f.agro@unicampus.it (F.E.A.); 2 Unit of Anesthesia, Resuscitation, Intensive Care and Pain Management, ASST Gaetano Pini, P.zza A. Ferrari 1, 20122 Milano, Italy; romualdodelbuono@gmail.com

**Keywords:** shoulder, regional anaesthesia, triple monitoring, opening injection pressure, interscalene brachial plexus block, arthroscopic shoulder surgery, ultrasound

## Abstract

Nerve injury is a feared complication of peripheral nerve blockade. The aim of this study was to test the effectiveness of a triple monitoring (TM), i.e., a combination of ultrasound (US), nerve stimulation (NS) and opening injection pressure (OIP) during interscalene brachial plexus block (IBPB) for surgery of the shoulder. Sixty patients undergoing IBPB for shoulder arthroscopy received TM. BSmart^®^, an inline injection device connected to a 10 mL syringe, was used to detect OIP during IBPB. Nerve stimulation was set to 0.5 mA to rule out any motor response, and if OIP was below 15 PSI, 10 mL of local anaesthetic was injected under US guidance between the C5 and C6 roots. The main outcome was the ability of TM to detect a needle–nerve contact. Other outcomes including the duration of IBPB; pain during injection; postoperative neurologic dysfunction. Triple monitoring revealed needle–nerve contact in 33 patients (55%). In 18 patients, NS evoked motor responses despite first control with US; in a further 15 patients, BSmart^®^ detected an OIP higher than 15 PSI, despite the absence of motor response to NS. Mean duration of IBPB was 67.2 ± 5.3 seconds; neither pain during injection nor postoperative neurologic dysfunctions were detected. Clinical follow up excluded the presence of postoperative neuropathies. Triple monitoring showed to be a useful and feasible tool while performing IBPB for arthroscopic shoulder surgery. Future studies will be needed to confirm our findings.

## 1. Introduction

Interscalene brachial plexus block (IBPB) is one of the most performed anaesthetic techniques to manage perioperative pain in shoulder surgery [1]. This approach fully blocks the sensitive innervation of the anatomical area interested by the surgery, permitting the use of regional anaesthesia as the main anaesthetic technique and reducing postoperative pain, nausea, opiates consumption and the patient’s hospital length of stay [2,3].

Nerve injury with consequent neurologic dysfunction is a feared, although uncommon, complication of peripheral nerve blockade [4]. Usually, most neurologic symptoms are transient, but they may occasionally cause a permanent disability with a negative impact on the patient’s day-to-day quality of life, resulting in chronic dysfunction, higher healthcare costs, and possible legal issues.

Recent literature reports that neurological symptoms associated to nerve blocks occur in up to 2.2% of patients at 3 months, 0.8% at 6 months and 0.2% at 1 year [5].

While performing nerve blocks, interpretation of the available information is pivotal in guiding the operator to place the needle tip in the right position, in order to prevent these complications.

The aetiology of nerve damage is multifactorial, and it is linked to the chemical neurotoxicity of the anaesthetic drugs, mechanical damage as a consequence of needle–nerve contact and neural fascicles break due to the high intraneural injection pressure [6,7]. Over the past years, ultrasound (US) and neurostimulation (NS) represented the “gold standard” of monitoring techniques for nerve blockade [8]. The concept of “opening injection pressure (OIP)” and its ability to detect a needle–nerve contact and intraneural injection has been recently introduced [9,10,11,12]. Opening injection pressure is defined as the pressure to be applied to the syringe piston that is necessary to win the resistance at the tip of the needle and make the flow start. This pressure is higher or equal than 15 per square inch (PSI) when the needle is located intraneural, while an OIP less then 15 PSI ensures an extrafascicular injection).

No studies have totally clarified how and whether it is feasible to introduce OIP in daily clinical scenarios and if it consistently impacts on block safety when compared to the double monitoring provided by US and NS.

The aim of this study was to verify the clinical applicability, efficacy and sensitivity given by the triple monitoring (TM) method, a combination of US, NS and OIP, during the execution of the IBPB with a lateral approach for shoulder arthroscopy surgery and its ability to prevent an unsafe intraneural injection.

## 2. Methods

This was a single-centre, prospective, single-arm study performed at the University Hospital Campus Bio-Medico of Rome. Hospital ethics committee approval was obtained before starting the patients’ enrolment (protocol number: 09.16 TS).

Patients’ inclusion criteria were age >18 years old, candidate for arthroscopic shoulder surgery, no contraindication to IBPB, American Society of Anesthesiologists (ASA) physical status I–III, signed informed consent.

Patients’ exclusion criteria included ASA IV, allergy to local anaesthetics, pre-existing infection at the injection site, contraindication to interscalene block (such as contralateral hemi-diaphragmatic paralysis), upper limb neurological deficit, informed consent not signed.

**Figure 1:**
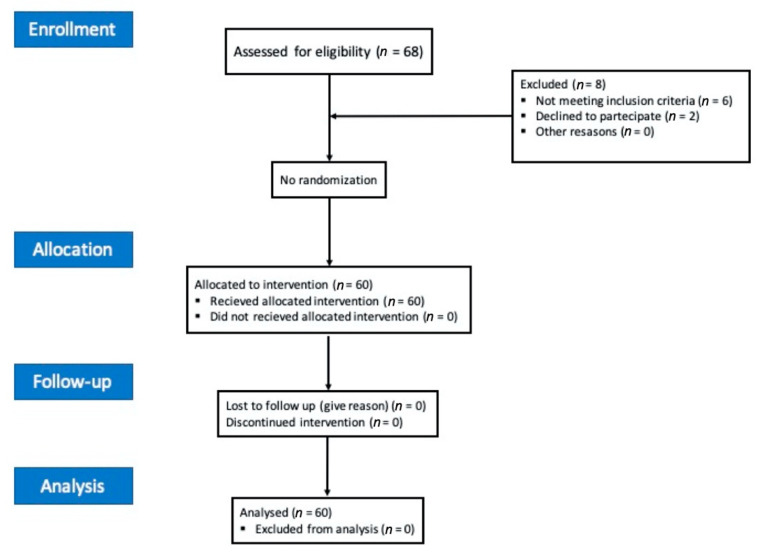
Consolidated Standards of Reporting Trials (CONSORT) 2010 flow diagram.

Sixty patients undergoing arthroscopic shoulder surgery under IBPB were assessed for eligibility in the study from January to July 2020, after informed consent was signed (Figure 1).

Before carrying out the IBPB, every patient was premedicated with midazolam 0.025 mg/kg and fentanyl 1 mcg/kg e.v.

**Figure 2:**
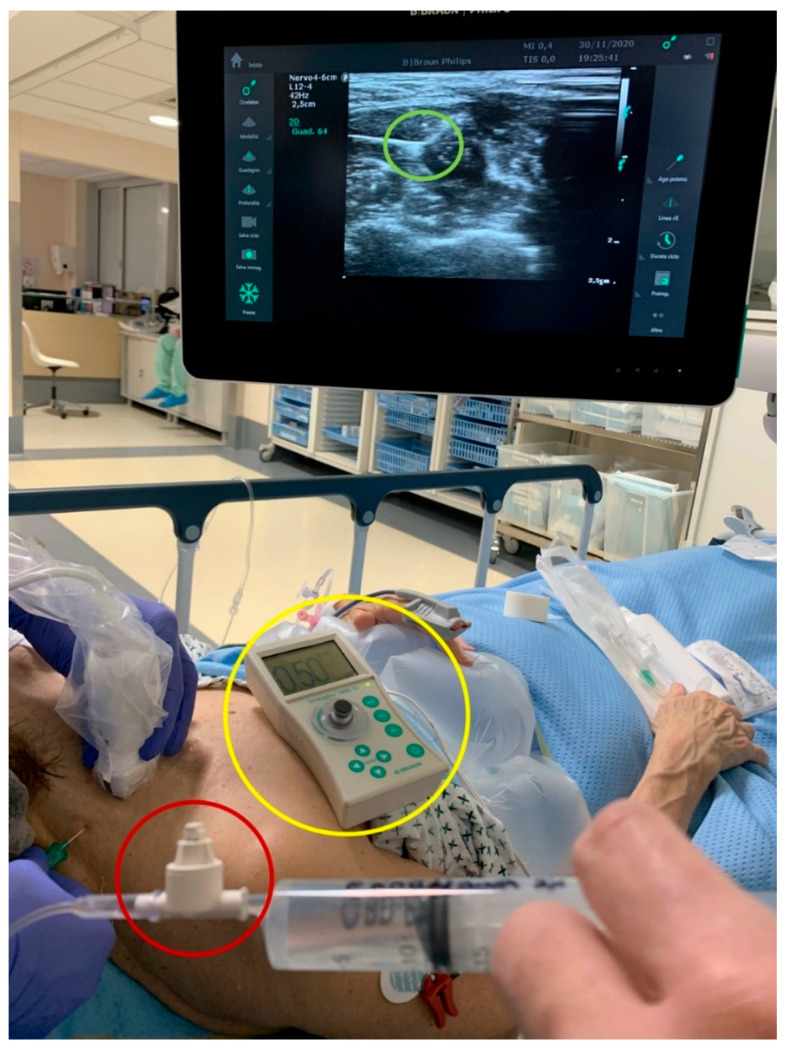
The triple monitoring devices. Green circle: ultrasound guidance; yellow circle: nerve stimulator; red circle: opening injection pressure monitoring device (BSmart^®^).

The TM during the execution of the IBPB was performed using with the following devices (Figure 2):

- An ultrasound machine (Sonosite M-Turbo, Sononite, Bothell, WA, USA) with multifrequency linear probe (8–12 MHz);
- A nerve stimulator (Stimuplex TM HNS 12, B. Braun Medical, Melsungen, Germany) set at 0.5 mA (2 Hz, 0.1 ms);
- BSmart^®^ (B. Braun Medical, Melsungen, Germany), an injection pressure monitoring system designed to avoid accidental intraneural injection. This device must be placed between the syringe and the needle extension tubing. It consists of a piston with three different coloured bands indicating three different levels of injection pressure (white: <15 psi; yellow: 15–20 psi; red: >20 psi). An OIP ≥ 15 PSI is suggestive of intraneural injection.

After signing the informed consent, the patients were asked to rotate the head on the contralateral side with respect to the shoulder to be operated, then the interscalene BPB was approached

**Figure 3:**
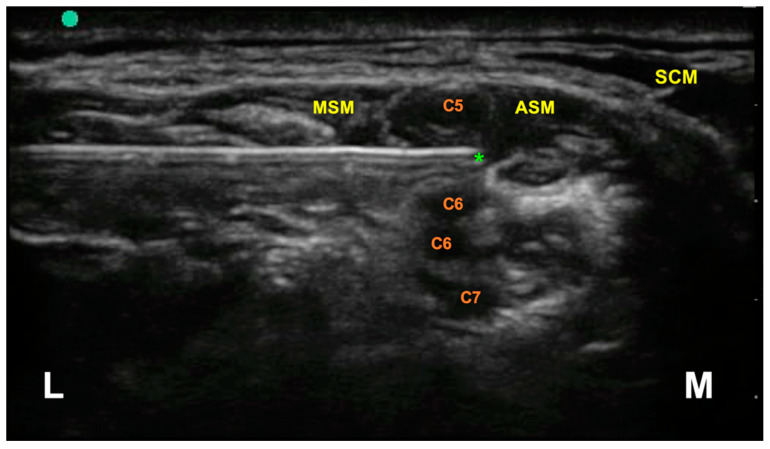
Interscalene brachial plexus, ultrasound anatomy. The asterisk indicates the target of the needle tip during the block, located between the C5 and C6 nerve roots. MSM = medial scalene muscle; ASM = anterior scalene muscle; SCM = sternocleidomastoid muscle; L = Lateral; M = Medial.

With sterile materials and technique, the needle (Stimuplex Ultra 360—50 mm, B. Braun Medical, Melsungen, Germany) was introduced in a lateral–medial direction through the posterior margin of the medium scalene muscle, maintaining a US in-plane approach. In this way, it was possible to control in real time the needle tip which was placed between C5 and C6 brachial plexus roots (Figure 3).

With the needle tip in place, the second control was performed through NS, verifying the absence of a motor response at a stimulation intensity ≤0.5 mA; in case of a motor response, the needle tip was repositioned under ultrasound guidance [13,14].

Lack of motor response to NS allowed the injection of the local anaesthetic in a “single-shot” modality.

The final control was the OIP, detected by the BSmart^®^. The injection was performed only if the pressure detected was less than 15 psi. If the OIP was equal or higher than 15 psi, the injection was suspended: the procedure was recovered after the tip of the needle was pushed back by 1 mm, and a new negative neurostimulation was obtained. Then, the procedure was completed only when the OIP was less than 15 psi. The coexistence of correct US-guided needle tip visualization, an absence of motor response at NS and an OIP < 15 psi suggested to us to exclude a needle–nerve contact or an intraneural injection.

**Figure 4:**
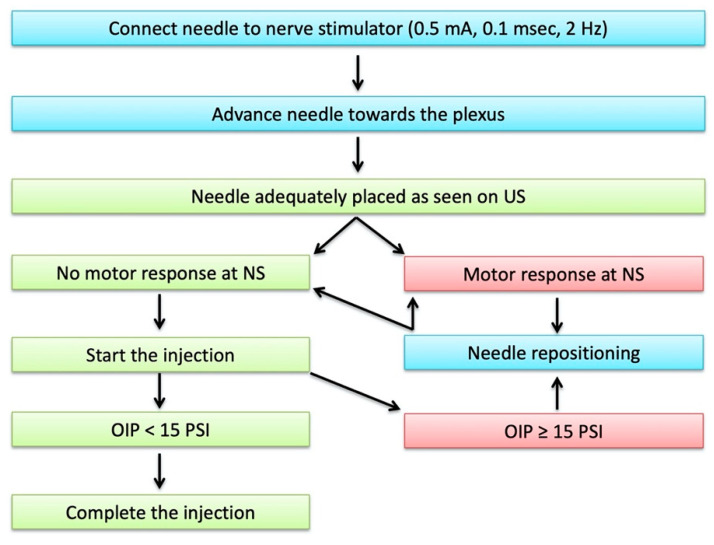
The triple monitoring flowchart. NS: nerve stimulation; US: ultrasound; OIP: opening Injection pressure.

The TM flowchart is summarised in Figure 4.

All patients received 10 mL of ropivacaine 0.75% (37.5 mg) and all the procedures were performed by the same anaesthetist, experienced in regional anaesthesia.

The following data were recorded in order to assess the TM efficacy: duration of IBPB from needle insertion to end of injection; block onset and duration; presence of any pain during injection; presence of postoperative neurologic dysfunctions.

Possible neurological complications in the postoperative period were investigated through follow up at 24 h, 7 days and 30 days. The following outcomes were evaluated for each timepoint: sensitive and motor function and the presence of any dysesthesia or neurological deficits pertinent to the nerve structures involved by the block. Sensitive function was evaluated by the pin-prick test at the deltoid area, while motor function was evaluated by the ability to adduct and flex the arm.

### Statistical Analysis

The values of categorical variables are expressed as a number and percentage. The parametric distribution of continuous variables are expressed in mean ± standard deviation (SD) and were evaluated using Shapiro–Wilk normality tests.

McNemar’s test was used to analyse the difference in categorical variables related to the different monitoring systems (with and without OIP). The statistical significance level was set for *p* = 0.05 values. All statistical analyses were carried out using State/IC 12.1 software (StataCorp, College Station, TX, USA).

## 3. Results

Sixty patients undergoing IBPB for shoulder arthroscopy received TM during the procedure.

Table 1 summarises patients characteristics and the main outcomes of the study.

**Figure 5:**
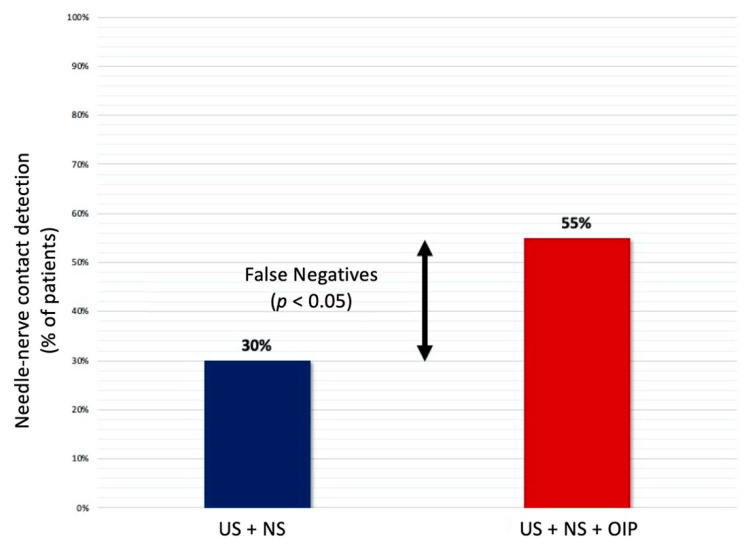
Needle–nerve contact detection.

Needle–nerve contact was detected with NS in 18 patients (30%) despite the first control with US. Moreover, adding the BSmart^®^ allowed for the detection of an OIP ≥ 15 psi (positive value for intraneural needle tip positioning) in a further 15 patients who had a negative NS response for needle–nerve contact (Figure 5).

Therefore, the triple monitoring (US + NS + OIP) was able to detect an unsafe needle placement in 33 patients out of 60 (55%). This difference in the rate of detections, analysed using McNemar’s test, was found to be statistically significant (*p* = 0.038).

The mean time for the interscalene block execution was 67.2 s ± 5.3 (SD).

The mean time of blockade onset was 13 ± 5.2 min, verified with the pin-prick test at the deltoid region.

The effectiveness of the block was also confirmed by intraoperative vital parameters stability, which lasted from the surgical incision until the end of the operation; moreover, no additional analgesic drugs were administered both intraoperative and in the Postanaesthesia Care Unit (PACU).

The mean block duration time was 8.5 ± 1 h for motor function (adduction of the arm) and 10 ± 2 h for sensitivity.

Neither pain during injection nor postoperative neurologic dysfunctions were detected in any patients.

Finally, clinical-anamnestic follow up of patients at 24 h, 7 days and 1 month after surgery excluded the presence of short- and/or medium-to-long-term or delayed neuropathies.

## 4. Discussion

Arthroscopic shoulder surgery is usually performed under IBPB to successfully control perioperative pain [3]. This regional anaesthesia technique has a long history, and its safety has improved in the last few decades thanks to the spread of US guidance [15,16].

Nerve injury is an uncommon complication of nerve blocks, but it may occasionally lead to serious consequences for the patient [5].

Cadaveric studies clearly demonstrated that a high OIP (≥15 PSI) may be associated with intraneural needle positioning [17]. Combining the US and the NS guidance together with the OIP measuring could help to avoid accidental intraneural local anaesthetic injections, but its ability to improve safety in IBPB and its applicability in daily practice still remain unclear.

In this study, we performed IBPB with TM in 60 patients who underwent arthroscopic shoulder surgery. Ultrasound control was firstly improved with NS to detect unsafe needle tip positioning; then, OIP was monitored, adding a third level of control.

Analysing the ability to identify a possible contact between needle tip and nerve, an unsafe needle positioning was found in 18 patients (30%) adding NS control to US guidance; TM, on the other hand, detected an unsafe injection in a total of 33 patients (55%).

This means that the addition of OIP monitoring using the BSmart^®^ device allowed for the identification of a further 15 needle–nerve contacts (25% of total patients) missed by the first two control stages (US + NS). This difference in the detection rate was found to be statistically significant (*p* < 0.05).

Multiple previous in vivo clinical studies have demonstrated the efficacy of OIP to identify needle–nerve contact [11,18]; however, this is the first study that demonstrates the superiority of triple monitoring (US + NS + OIP) against US guidance plus NS control in detecting needle–nerve contact. The presence of false negatives at the NS control is consistent with the results reported by Tsay et al. [19], who stated that motor response to the NS could sometimes be absent even if the needle is placed intraneural with low current intensity.

Our results suggest that this triple localisation technique can be used effectively to identify and, subsequently, reduce needle-to-nerve contact.

Moreover, our data show that performing three different methods of nerve localisation does not require a significant additional time and, definitely, it does not delay surgery. Not by chance, the block execution time was similar to the one showed by other studies without the use of triple monitoring [13,20], while the block onset time was consistent with the one found in other experiences using similar dosage of local anaesthetic [21,22].

No neurological injuries or symptoms were found after 24 h, 7 days and 30 days follow up, confirming the absence of nerve injuries caused by the IBPB performed under triple monitoring.

However, this study had several limitations. First of all, it was a single-arm study without a control group. Moreover, the number of patients included was limited, and this affected the probability of recording postoperative neurological injuries after IBPB, the incidence of which is estimated to be up to 2.2% in the general population undergoing shoulder surgery [5]. Furthermore, greater consideration should be given to the fact that TM did not avoid inadvertent needle–nerve contact, which occurred in over half of cases, even if it was unlikely to cause nerve damage alone, without injecting. However, TM prevented from the injection of local anaesthetic during the needle–nerve contact, which could potentially cause nerve damage.

Controlled trials with larger samples could clarify the efficacy of triple monitoring in improving safety and efficacy of IBPB for shoulder surgery without affecting block success.

## 5. Conclusions

This prospective single-arm study suggested that triple monitoring is a feasible technique that effectively identifies needle-to-nerve contact, which may potentially have implications for reducing nerve injury during IBPB for arthroscopic shoulder surgery. Ultrasound, NS, and OIP are synergistic, allowing for the optimal needle tip placement and preventing intraneural injection of local anaesthetic.

Randomized controlled trials with larger samples are needed to confirm our findings.

## Figures and Tables

**Table 1:** Patients’ characteristics and main outcomes.

**Patients’ Characteristics and Main Outcomes**	
Number of patients	60
Sex (M/F)	24/36
Age (Mean ± SD), years	59.9 ± 5.3
BMI (Mean ± SD), kg/m^2^	26.4 ± 5.9
Time for nerve block execution (Mean ± SD), s	67.2 ± 5.3
Pain during block execution	none
Needle–nerve contact detection, *n* (%) - US + NS - US + NS + OIP	18 (30%) 33 (55%)
Block onset time (Mean ± SD), min	13 ± 5.2
Motor block duration (Mean ± SD), hrs	8.5 ± 1
Sensitive block duration (Mean ± SD), hrs	10.2 ± 2
Postoperative neurological complications	none

BMI: Body Mass Index; SD: Standard Deviation; US: Ultrasound; NS: Nerve Stimulation; OIP: Opening Injection Pressure.

## Data Availability

The data presented in this study are available on request from the corresponding author. The data are not publicly available due to restrictions regarding patients privacy.
